# Using a bio-scanner and 3D printing to create an innovative custom made approach for the management of complex entero-atmospheric fistulas

**DOI:** 10.1038/s41598-020-74213-7

**Published:** 2020-11-16

**Authors:** Virginia Durán Muñoz-Cruzado, Francisco José Calero Castro, Andrés Padillo Eguía, Luis Tallón Aguilar, José Tinoco González, Juan Carlos Puyana, Felipe Pareja Ciuró, Javier Padillo-Ruiz

**Affiliations:** 1 Division of General Surgery, Virgen del Rocío University Hospital, Av Manuel Siurot S/N, 41013 Seville, Spain; 2 University of Seville, Seville, Spain; 3 Division of Surgery, Critical Care Medicine and Clinical Translational Science, University of Pittsburgh, Pittsburgh, USA

**Keywords:** Biotechnology, Diseases, Gastroenterology

## Abstract

Enteroatmospheric fistulae are challenging clinical conditions that require surgical expertise and that can result in chronic debilitating conditions placing the patient in a vicious cycle characterized by non healing wounds and malnutrition. They are a complex entity that presents great variability depending on the number, shape, and size of the fistulous orifices, their debit, and the dimensions of the wound. This means that, at present, there is no device that adapts to the anatomical characteristics of each patient and manages to control the spillage of intestinal effluvium from the wound. The aim of this study is to describe the manufacturing technique and to assess the preliminary results of a custom device designed through bioscanner imaging and manufactured using 3D printing for use with negative pressure wound therapy (NPWT) in the management of enteroatmospheric fistula. A proof of concept is given, and the design of the device is presented for the first time. After obtaining images of each fistula with a bioscanner, a personalised device was designed for each patient by 3D printing shape of a prism and a hollow base, taking into account the dimensions of the fistulous area in order to perform a floating ostomy to isolate the wound from the debit enteric. The polycaprolactone (PCL) device was placed including inside the fistulous surface and surrounding it with the NPWT system in order to accelerate wound healing.

## Introduction

Enteroatmospheric fistula is defined as the communication between the intestinal lumen and the surface of an open abdominal wound^1–6^. It occurs in 10% of patients who have an open abdomen due to treatment for peritonitis or compartment symdrome^5^ causing 40% mortality^4^. The main challenge presented by this pathology is the local control of the fistula, that is, to ensure that the intestinal content that is poured through the fistula does not come into contact with the wound, since the chemical burn and the contamination produced by the Intestinal material prevents effective granulation an adequate healing of the surrounding tissue.

The local control of the fistula presents a wound care challenge due to the enormous variability of this entity: the size of the wound, the position and number of fistulous orifices, intestinal segments involved, and the volumen of intestinal output, etc. These many factors result in complex management challenges and currently, due to the great anatomical variability of these fistulae there is not a unique device or solution that can be used to ensure adequate local control in this very heterogenous complication. typical of complex enteroatmospheric fistulae.

The ideal treatment is resection of the intestinal segment involved and an anastomosis with the reconstruction of the abdominal wall^1,2,4^. This treatment is not feasible at the time of diagnosis due to the peritoneal adhesions presented by the patients and the inflammation of the surrounding tissues; therefore, there is bridge therapy that is used for externalisation of intestinal fluids to avoid continuous contact with the wound, thus favouring the correct healing and granulation of the wound until definitive surgery can be performed^1,2,4^.

One of the alternatives of bridge therapy is negative pressure wound therapy (NPWT), which is applied to the wound surface to promote granulation. However, this therapy must be accompanied by a device that is capable of isolating the fistulous orifice of the NPWT, since if negative pressure is produced on the fistula, it would have untoward consequences and therefore perpetuating and even enlarge the fistulous surface.

Thus, different techniques have been described to isolate the wound from the faecal material and to provide correct granulation of the wound by combining NPWT with various devices that help establish a “floating stoma”^4^.

These therapies include artisanal and commercial devices intended to isolate the fistula.

However, the clinical variability and the various presentations of this pathology make the currently designed devices not valid for all types of fistulas, making personalised treatment of these fistulas necessary.

Given the special characteristics of the enteroatmospheric fistula that make personalised treatment necessary, we proposed the creation of a customised adapter, made of polycaprolactone (PCL) by 3D printing, for each patient and for each evolutionary moment based on the characteristics of the patient.

## Materials and methods

### Patient selection

From June 2017 to September 2019, all patients at our centre or derived from other centres that were over 18 years of age and diagnosed with enteroatmospheric fistula were prospectively preselected for the study. Exclusion criteria included the following: shapes or dimensions of the wound that did not allow for application of the device (for example, a fistula very close to the edge of the wound), clinical conditions of the patient that prevented placement of the device (hemodynamic instability or need for urgent surgery), and not signing the informed consent.

This study has been approved by the ethics committee (Research Ethics Committee of Seville). All experiments were performed inaccordance with relevant named guidelines and regulations. Informed consent was obtained from all participants and/or their legal guardians, even to publish the information and images in an online open-access publication.

In this period, five patients were shortlisted. Of these, four were included in the study; one patient was excluded because they did not sign the informed consent.

The clinical characteristics of the selected patients and the evolutionary changes of the fistula are described in Table 1.

**Table 1:** Baseline characteristics of the patient.

Patient	Age (years)	Initial cause	Time of evolution (days)	Intestinal segment	Daily debit (cc/24 h)	Diameters of the wound transverse/length/depth (cm)	Fistulous surface (cm^2^)	Number of fistulous holes	Fistula scheme
1	56	Septic complication of abdominal amputation due to anal canal epidermoid tumor (6 surgical interventions)	150	Yeyuno	800 (high)	14/12/2	42.41	4	
2	45	Acute Diverticulitis WSES IV (7 surgical interventions)	10	Terminal ileon	500 (medium)	13/16/6	18.28	3	
3	69	Duodenal perforation (2 surgical interventions)	7	Duodenum	100 (low)	3.a. Initial moment: 4/20/2 3.b. Change during therapy: 3/19/1	3.a. Initial moment: 0.78 3.b. Change during therapy: 0.75	1	
4	62	Late evisceration after intestinal obstruction due to bezoar (1 surgical intervention)	2	Yeyuno	300 (medium)	4.a. Initial moment: 9/15/2 4.b Change during therapy: 9/15/2	4.a. Initial moment: 1.1 4.b Change during therapy: 13.74	1	

*WSES* World Society of Emergency Surgery.

### Description of the intervention applied

#### Device design

**Figure 1:**
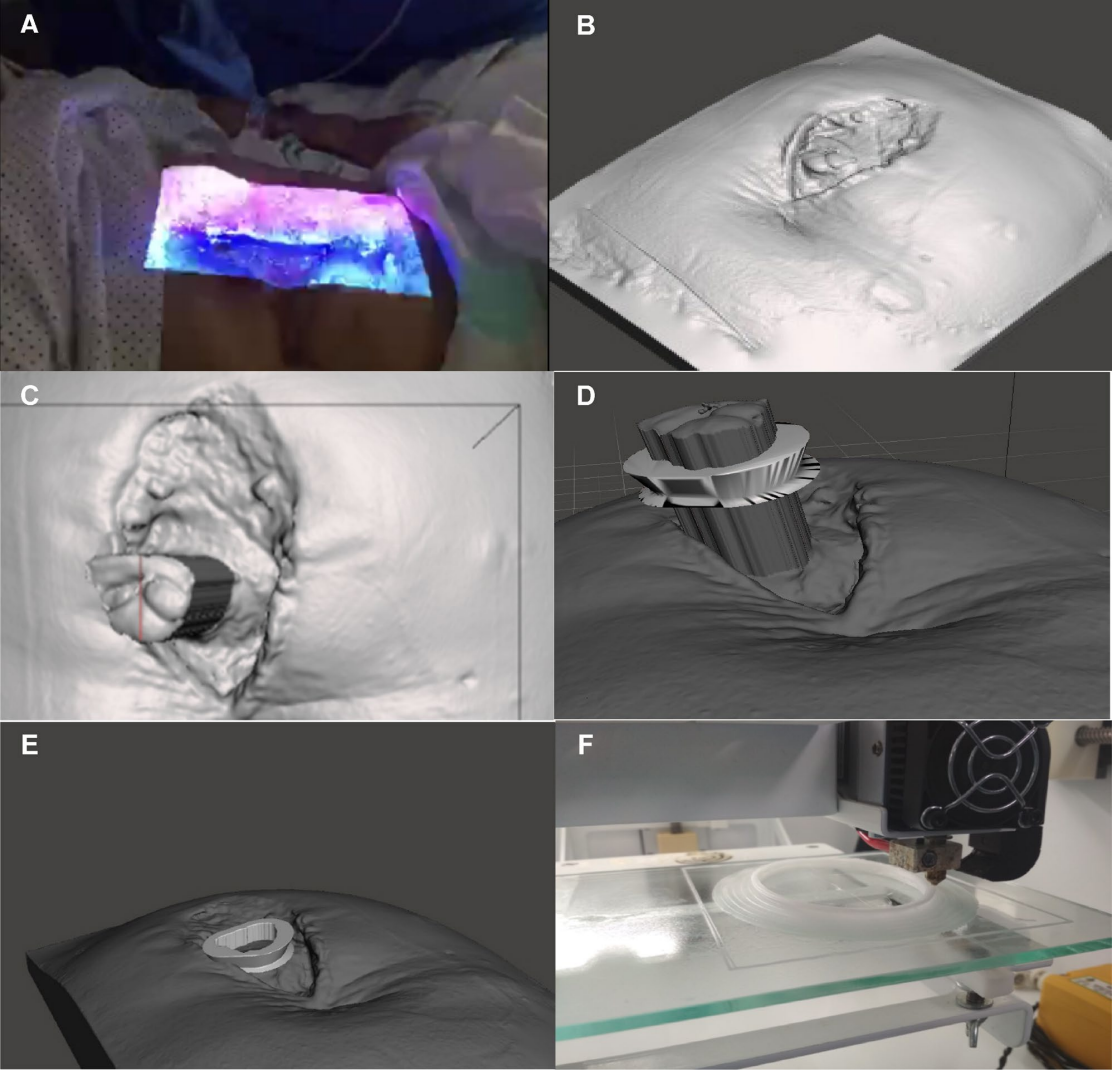
Process of using of bioscanning and 3D printing. (**A**) Process of taking pictures with the bioscanner. (**B**) Images obtained with the bioscanner. (**C**) Measurement of the exposed intestinal surface dimensions for device design. (**D**) Verification of the suitability of the prosthesis by extrusion of the fistulous surface. (**E**) Placement of the device on the image of the bioscanned wound to determine the correct adaptation to the patient. (**F**) 3D printing of the bioprosthesis.

The design of the adapter was personalised for each patient, taking into account the dimensions of the exposed intestinal surface of the enteroatmospheric fistula and the dimensions of the wound, and images of each patient were obtained through the bioscanner (Einscan pro+, 3D shining, China) (Fig. 1A,B). Once the precise dimensions of the fistulous surface were known, the device was designed using FreeCAD 0.16. software (FreeCAD Juergen Riegel, Werner Mayer, Yorik van Havre).

The design of the device was based on a prism with different shapes and a hollow interior. The intestinal surface that forms the fistula was inside the prism, and the abdominal wall wound was isolated from the intestinal contents, generating a floating ostomy when applying the NPWT system around the device. In order to correctly adapt to the NPWT system, protrusions were created at both ends of the device, which were exteriorised at the top, in contact with the adhesive plastics of the NPWT system, and at the bottom, in contact with the wound.

Each of these are personalised devices, and the characteristics of each design are described in Table 2. For patient 3 and 4, two different devices were designed due to the evolutionary changes that the wound had presented.

**Table 2:** Characteristics of each device.

Device	Shape	Device dimensions (cm)	Centre hole size (cm^2^)	Device height (cm)	Top output size (cm)	Lower output size (cm)	Bioscanner design	3D printed device
1	Oval	Major radius: 4.5 Minor radius: 3	42.41	2	1.5	1		
2	Rectangular base with two semicircles at the ends	Device base: 8 Major end radius: 1 Minor end radius: 1.5	18.28	2	1.5	1		
3.a	Circular	Radius: 0.941	2.78	1	0.3	0.3		
3.b	Oval	Major end radius: 3.394 Minor end radius: 2.555	2.2	1.5	1	1		
4.a	Circular	Radius: 0.643	0.566	1.5	0.3	0.2		
4.b	Triangular	Length: 6.330 Width:4.486	14.903	1.5	0.7	0.5		

Once the device was tailored to the fistulous surface with FreeCAD 0.16 software, it was exported in stl format. The scanned surface was imported into the Meshmixer software (Autodesk, San Rafael, EEUU) next to the device. The fistulous wound was selected and extruded in order to perform a Boolean operation in with intestinal surface is subtracted through the hole in the device. Finally, the ring-shaped device was placed on the image of the wound to verify the customisation and placement (Fig. 1C–E). Through the virtual simulation of the coupling of the prosthesis on the surface of the patient's wound, we can generate the tolerance margin so that the fistulous tissue is not pressed by the prosthesis and thus avoiding erosions around it.

#### Manufacturing

Once the designs were made, they were exported in an .stl file, which was read by Regemat 3D designer software, and .gcode files were created and sent to the 3D printer (Regemat 3D, Granada, Spain).

The impression was made with clinical grade PCL (99 filament 750 g 1.75 mm, 3D4makers, Haarlem, The Netherlands) with a solid fill pattern and a layer height of 0.35 mm. The head speed was 20 mm/s for the filling, and for the perimeter it was 10 mm/s. The printing flow was 1.8 mm/s at a temperature of 80 °C (Fig. 1F). After printing the device, a refinement phase is undertaken. This is done in order to create the best possible fitting between the device and the tissues surrounding the fistula. Due to the complex trajectory that the bioprinter must follow, it is sometimes difficult to obtain a perfectly smooth device that adapts to all edges of the wound.

The total time to carry out the complete process of generating the device is an average of 4 ± 0.45 h.

Prior to application in patients, each device was sterilised according to the standards of the centre for sterilisation of biomaterials.

Two devices of each design were manufactured. In the case of patient 3 and 4, due to the evolutionary changes that are characteristic of this pathology, two different devices were designed according to the needs of the patient and the evolutionary moment (device 3a, 3b, 4a and, 4b from Table 2).

#### Device placement

**Figure 2:**
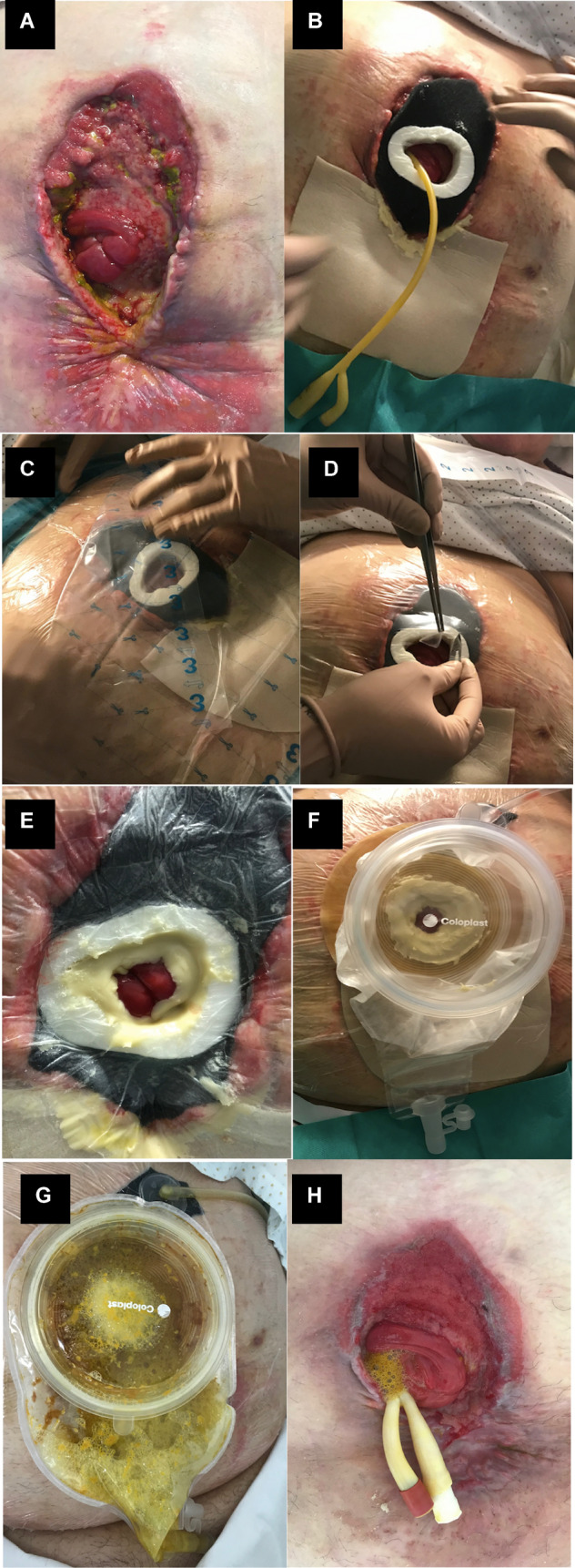
Device placement in combination with negative pressure therapy. (**A**) Initial status of patient 4. (**B**) Insertion of the prosthesis in the polyurethane sponge and placement on the wound. (**C**) Application of a plastic sheet to cover the device and the sponge. (**D**) Hole in the internal part of the prosthesis that allows the intestinal material to escape. (**E**) Application of the vacuum system and application of stoma paste to preserve the vacuum. (**F**) Placement of an ostomy bag. (**G**) Implanted device with observed outflow of enteric material through the central hole of the prosthesis, with collects the ostomy bag. Vacuum therapy maintained in the upper part of the wound without enteric content. (**H**) Final outcome of patient 4.

For application of the NPWT system, the abdominal wound was washed with physiological serum to eliminate possible remains of the intestinal discharge (Fig. 2A). A sponge was moulded to the shape of the abdominal wound of each patient, and inside it an opening the size of the device was made. In this opening, the custom adapter was placed, and the rest of the abdominal wound was covered with the sponge, leaving the intestinal surface inside the adapter (Fig. 2B).

To facilitate the adhesion of the NPWT adhesive film, we lined the device with transparent polyurethane adhesive dressings (Opsite, Smith and Nephew). Adhesive films were used to completely cover the wound and the sponge, and, subsequently, a hole was made in the adhesive film on the internal part of the prosthesis that allowed the intestinal material to escape (Fig. 2C,D).

The sponge was subsequently connected to the NPWT device at a pressure of − 125 mmHg. For correct sealing of the system, which maintains the vacuum, the angle formed between the internal walls of the adapter and the surface of the fistulous area was covered with sealing paste (Stomahesive, ConvaTec) (Fig. 2E). In this way, a floating ostomy was performed, where the intestinal surface of the fistula protrudes through the interior of the tailor-made adapter, leaving the abdominal wound completely separated from the intestinal discharge (Fig. 2F). Finally, an ostomy bag was placed in the system to collect the intestinal contents (Fig. 2G). The procedure was the same for each replacement and for each patient.

### Statistics analysis

The qualitative variables were expressed with absolute and relative frequencies, and the quantitative variables with mean and standard deviation, and median and interquartile range (RIC) if the distribution was not normal. Given the characteristics of the sample, a before-after study is proposed. The variables of interest collected. For this, analyzes of related samples were performed using the Student's T test for paired samples in those cases in which continuous variables behaved through a normal distribution. In the case of needing a nonparametric alternative to perform the contrast, the Wilcoxon test was used. It was accepted that the difference between the groups was statistically significant if p < 0.05. The data was processed and analyzed with the IBM SPSS v24.0 statistical package.

### Meeting presentation

An earlier version of this paper was presented at International Trauma and Emergency Surgery Week in Alicante (Spain) in December 2019.

## Results

Application of the designed device allowed for isolation of the wound from the intestinal contents, favoring granulation of the wound, reducing the need for cures, improving the comfort of the patient, and avoiding complications derived from discharge of the intestinal effluvium to the surgical area. The time of application of the therapy was 23, 8, 15, and 30 days for each of the consecutive patients.

In all cases, the need for weekly cures was reduced for all patients from an average of 31.5 ± 4.04 to 3.125 ± 0.48. During treatments prior to the placement of the device, the pads were continuously soaked in intestinal fluid, conditioning a continuous chemical abrasion of the skin surrounding the wound that caused itching and pain in patients. This continuous abrasion was eliminated with the placement of the device, which facilitated healing of the erosion at the edges of the wound, improved patient comfort, reduced medication requirements for the treatment of pruritus, and progressively decreased the daily needs of morphic chloride, with an average daily dose of 3 ± 1.15 ml until use of first level analgesic (Table 3).

**Table 3:** Results of the therapy.

Efficiency variable	Before therapy (average)	After therapy (average or median)	p
Number of weekly wound cures	31.5 ± 4.04	3.125 ± 0.48	0.001
Pruritus (visual analogue scale of pruritus)	6 ± 0.82	0 [0–2]	0.006
Pain (visual analogue pain scale)	6.5 ± 1.29	1.5 [0–4]	0.002
Daily dose of morphic chloride for pain (ml)	3 ± 1.15	0	0.014
Wound dimensions (cm)	Longitudinal: 14.25 ± 4.79	Longitudinal: 12.87 ± 4.94	0.06
	Transverse: 11.5 ± 5.07	Transverse: 6.5 ± 3.70	0.14
	Depth: 3 ± 2	Depth: 1.25 ± 0.5	0.16

Association with the NPWT encouraged progressive granulation of the surrounding tissue, reducing the dimensions of the wound by an average of 1.38 ± 0.95 cm in longitudinal diameter, 5 ± 4.32 cm in transverse diameter, and 1.75 ± 1.5 cm in depth (Table 3).

In patient 1, the final closure of the fistula occurred after application of the therapy. The final results of “ostomisation” (the creation of a healthy surface of surrounding tissue that best limits the actual orifice) were achieved in patients 3 and 4, transforming a wound with poor initial management into an intestinal segment surrounded by enough tissue to adapt an ostomy bag (Fig. 2H). In patient 2, the granulation initially obtained with therapy provided an excellent substrate for the application of an anterolateral thigh flap to cover the wound defect with which the closure of the fistula was obtained.

## Discussion

To our knowledge, this is the first study that describes the development of a standardized methodology, for the design and manufacturing of a customized prostheses in patients, who suffer complex enteroatmospheric fistula. Specifically, obtaining images for 3D prostheses using a bioscan is a novel method that has not been previously described in the literature.

Enteroatmospheric fistulas are characterised by extensive clinical variability in their presentation. To correctly define the type of fistula, we must know the amount of daily intestinal effluvium that is collected through it (high, > 500 ml/day; medium, 200–500 ml; or low, < 200 ml/day), the location in the abdominal cavity (superficial or deep), the intestinal tract that is involved, and the number of fistulous holes that we find open on the surface of the wound. Depending on these parameters, a great diversity of fistulas can be described, which means that in each patient we have to propose personalised treatment adapted to the characteristics of the fistula^4^.

There are two objectives in the treatment of these patients: definitive closure of the fistula or ostomisation of the fistula, that is; getting the tissue surrounding the intestinal surface to granulate and epithelize so that we can adapt a conventional ostomy bag. The rate of spontaneous closure of the fistula varies depending on the cause and the amount of daily intestinal effluvium^7–9^. Since the enteroatmospheric fistula is an exposed hole in the intestinal lumen, without skin or soft tissue covering it, the chances of spontaneous closure are very low. In our case, in patient 3 there was closure after the application of therapy. An adequate nutritional status, a smaller fistulous surface compared to the other patients (although, not less than 1 cm) and non-malignancy of the process which leads to the development of the fistula, contributed to its closure. However, success of the therapy in not transmitting negative pressure at the level of the exposed intestinal surface that is included inside the prosthesis is essential for closure.

In 2002, for the first time, Subramainam et al.^10^ proposed the idea of a "floating stoma" for such isolation. These authors described a device that consisted of protection of the wound with an intestinal lining bag from the surroundings of the fistula to the outer edges of the wound and over it, an ostomy bag that collects the intestinal contents of the fistula. Subsequently, this idea has been perfected with the introduction of NPWT in the management of large wounds. To date, multiple devices have been described. The bottle nipple was one of the first devices described^11^. Other devices described by Verhaalen et al.^12^ are a ring with polyurethane foam covered with adhesive, forming an impermeable barrier for the isolation of the fistula. Other authors have used adapters of standard size that manage to isolate the intestinal discharge from a soft thermoplastic elastomer. These devices are used to avoid pressure on the underlying surface when combined with the NPWT to perform a floating stoma^13,14^.

The methods described to date, whether commercial or conventional devices, do not take into account the different shapes and sizes that the open intestinal surface on the wound can present in an enteroatmospheric fistula, which, as we have commented previously, offers wide clinical variability. The fact that the description of numerous devices for local control of fistula exists in the literature is nothing more than a sign that there is currently an important clinical problem to be solved, which requires personalised therapy adapted to the needs of each patient.

Therefore, we believe that the personalisation of a device that adapts to the patient's own characteristics is essential. For the first time, our work presents the design, creation, and placement of an individualised device for the treatment of enteroatmospheric fistula.

Today, personalised treatment can be easily achieved thanks to 3D printing, which allows for the creation of devices through assisted design^7^. The use of 3D printing in medicine can be applied in several areas, ranging from surgical planning in traumatology or urology^15–17^ and the creation of orthoses or splints^18^ to tissue engineering and regenerative medicine for the creation of tissues from hydrogels and polymers^19^.

Obtaining images using a bioscanner for the design of 3D printed devices is pioneering since, to date, other prostheses have been made using images taken by computerised tomography or magnetic resonance imaging. This method of imaging is totally harmless to the patient, does not emit radiation, and allows for the accurate capture of images by avoiding the movements of the patient's breathing or intestinal peristalsis in the fistula. The image obtained in three dimensions allows for its movement in the three dimensions, which will allow us to delimit, in a more precise way, the relationship between the exposed intestinal surface and the rest of the wound. The novelty of this study is the introduction of an integrated architecture, using a 3D bioscann as a innovative strategy to acquire superficial images. These images allow to obtain anatomical information to build a virtual wound. We used a CAD program to create custom prosthesis. Subsequently, a 3D printing technique was implemented to manufacture perfectly fitting wound devices.

The main objective of the device generated for each patient was the isolation of the intestinal contents. To allow for this, a prism-shaped design was made with a hollow interior. To allow for good grip on the NPWT system, protrusions were created at the ends, which will end up in the prism wall, creating a kind of cone. However, the projection that is in contact with the abdominal wall has a smaller surface area. Therefore, there is a greater surface area of the abdominal wall in contact with the NPWT system. Our design resembled that created by Jannasch^9^, with a difference in individualisation and conical morphology in our device for adaptation to the NPWT system. To ensure the proper adaptation of the prostheses to the wound, the larger the size of the device, the less the surface of the wound will be in contact with the negative pressure therapy. This is critical to generate healthier and faster granulation of the surrounding tissue. Therefore, the size of device edges constitutes the minimum required to support the placement of negative pressure therapy in each case. Through the virtual simulation of the coupling of the prosthesis on the surface of the patient's wound, we can generate the tolerance margin so that the fistulous tissue is not pressed by the prosthesis and thus avoiding erosion of the area.

For manufacture of the device, PCL was used, which is one of the most used materials for 3D printing. It is a biocompatible material with good histological behaviour, and it has been used in many devices approved by the Food and Drug Administration (FDA) of the United States of America^20–26^. Previous studies have demonstrated good fibroblastic proliferation and mesenchymal stem cells; that is, the behaviour of the material is ideal for healing^20–26^. PCL is being developed with other biocompatible materials such as hydrogel or bioglass to form hybrid materials for use in medical devices^27,28^, this represents an opportunity for improvement for devices that we should explore in the future.

The synergy between the NPWT and the custom 3D printed adapter resulted in wound isolation of all patients for 24–48 h, without associated complications or leaks. After this period, the system was replaced to prevent the wound granulation tissue from integrating into the NPWT system sponge. A watertight system was achieved, which isolated the intestinal contents of the wound and the stimulation of the growth of the granulation tissue, ostensibly improving the comfort of the patient.

However, the management of these patients remains complex, especially at the local level, and placement of the device has not been without complications. Achieving and maintaining the sealing of the device is not simple; it requires deep knowledge of the characteristics of the fistula and the material with which it works. One of the main problems we have encountered is that the PCL material does not adhere properly to the NPWT system adhesives. To solve this problem, we lined the device with transparent polyurethane adhesive dressings (Opsite, Smith and Nephew) so that we would achieve an adherent surface on the adhesives of the NPWT system. Other alternatives could be a coating with a material that allows for adhesion or the use of other biomaterials that can be used in 3D printing. One of which is polylactic acid (PLA); however, this material is pending approval by the FDA of the United States of America.

This study was designed as a pilot study. We are aware of the technical limitations of the study and the small number of patients, and we continue to work on the development of clinical trials that include a larger study population to corroborate these results.

## Conclusions

After the initial development of this proof of concept, we conclude that the use of the bio-scanner and 3D printing in order to create a personalized device that fits the characteristics of the patient's wound is feasible and offers promising results in the management of entero-atmospheric fistulas. Subsequent studies with a greater number of patients will be necessary to form more consistent conclusions.
